# The rapid appearance of homostyly in a cultivated distylous population of *Primula forbesii*

**DOI:** 10.1002/ece3.9515

**Published:** 2022-11-18

**Authors:** Cai‐Lei Liu, Yin Jia, Yi‐Feng Li, Yuan‐Fen Xiang, Yuan‐Zhi Pan, Qing‐Lin Liu, Ke‐Hang Ma, Xian‐Cai Yin

**Affiliations:** ^1^ College of Landscape Architecture Sichuan Agricultural University Chengdu China

**Keywords:** distyly, floral scent, homostyly, *Primula*, pollinator visitation

## Abstract

Evolutionary breakdown from rigorous outbreeding to self‐fertilization frequently occurs in angiosperms. Since the pollinators are not necessary, self‐compatible populations often reduce investment in floral display characteristics and pollination reward. *Primula forbesii* is a biennial herb with distribution restricted to southwest China; it was initially a self‐incompatible distylous species, but after 20 years of artificial domestication, homostyly appeared. This change in style provides an ideal material to explore the time required for plant mating systems to adapt to new environmental changes and test whether flower attraction has reduced following transitions to selfing. We did a survey in wild populations of *P. forbesii* where its seeds were originally collected 20 years ago and recorded the floral morph frequencies and morphologies. The floral morphologies, self‐incompatibility, floral scent, and pollinator visitation between distyly and homostyly were compared in greenhouse. Floral morph frequencies of wild populations did not change, while the cultivated population was inclined to L‐morph and produced homostyly. Evidence from stigma papillae and pollen size supports the hypothesis that the homostyly possibly originated from mutations of large effect genes in distylous linkage region. Transitions to self‐compatible homostyly are accompanied by smaller corolla size, lower amounts of terpenoids, especially linalool and higher amounts of fatty acid derivatives. The main pollinators in the greenhouse were short‐tongued *Apis cerana.* However, homostyly had reduced visiting frequency. The mating system of *P. forbesii* changed rapidly in just about 20 years of domestication, and our findings confirm the hypothesis that the transition to selfing is accompanied by decreased flower attraction.

## Graphical abstract

Under the reproductive pressure, the mating system of *Primula forbesii* will rapidly change from self‐incompatible distyly to self‐compatible homostyly in just 20 years. Furthermore, with the transition from outcrossing to self‐pollination, plants will reduce their investment in flower display traits.

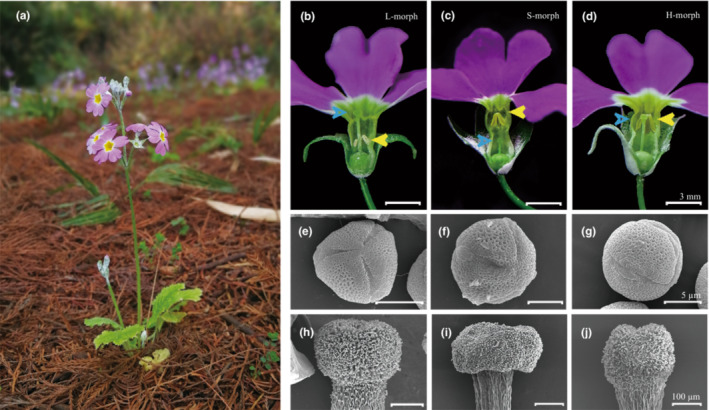

## 1 INTRODUCTION

The evolutionary transition from obligate cross‐pollination to autonomous self‐pollination frequently occurs among angiosperms (Igic et al., 2006; Shao et al., 2019; Zhong et al., 2019). In numerous families, the shift of mating system will affect the biodiversity, the response to selection, the evolution of floral signals and rewards, and the population structure (Charlesworth & Charlesworth, 1979; de Vos et al., 2014; Sicard & Lenhard, 2011; Yuan et al., 2017; Zeng et al., 2022). This transition has a profound ecological, evolutionary, and genetic influence on plant populations because it leads to reproductive isolation and subsequent speciation (Wright et al., 2013), which are interesting to biologists since Darwin's seminal research (Darwin, 1877; Yuan et al., 2019; Zhang et al., 2021; Zhou et al., 2017). Of special value for investigations of mating system changes are lineages that include outcrossing and self‐pollination, which provide a valuable opportunity to determine the selection forces and evolutionary consequences of selfing transitions.

A paradigmatic model for studies of shift from outcrossing to selfing is the evolutionary breakdown of the heterostyly to homostyly. Heterostyly is a genetically controlled floral polymorphism and includes two (distyly) or three (tristyly) floral morphs that differ reciprocally in stigma and anther position (Barrett, 1992; Darwin, 1877; Lloyd & Webb, 1992) and has arisen independently in at least 28 angiosperm families and 199 genera (Barrett, 2019). The major type of heterostyly is distyly, in which the flowers of one morph have a high stigma and low anthers (long style morph, L‐morph), while the other morph has a low stigma and high anthers (short style morph, S‐morph; Eckert & Barrett, 2008). Heterostylous plants possess heteromorphic self‐incompatibility, which prevents self‐ and intra‐morph pollinations and polymorphism in ancillary traits, such as dimorphism of pollen grains and stigma papillae (Barrett & Cruzan, 1994). Heterostyly is considered a classic model for promoting pollen dispersal efficiency and avoiding the harmful influences of inbreeding depression (Barrett, 1992, 2019; Brys & Jacquemyn, 2015; Ganders, 1979). However, the breakdown of sexual polymorphism frequently occurs in numerous heterostylous families, giving rise to self‐compatible homostyly (H‐morph) with stigma and anther at the same level (Brys & Jacquemyn, 2015; Darwin, 1877; Li & Johnston, 2001; Zhong et al., 2019; Zhou et al., 2017). Homostyly is also related to two floral morphs, namely the “long homostyles,” with high‐level stigma and anthers; or “short homostyles,” with low‐level stigma and anthers. The long homostyles are more common in nature for preferential spread and fixation (Charlesworth & Charlesworth, 1979; Yuan et al., 2017).

The hypothesis of reproductive assurance explains the occurrence of homostyly in heterostylous populations (Darwin, 1876), in which due to the unreliable service of pollinators and the limited number of pollen donors in extreme environments or colonizing episodes, the transition to self‐pollination is conducive (Brys & Jacquemyn, 2015; Busch & Delph, 2012; Moeller, 2006; Shao et al., 2019; Yuan et al., 2017; Zhang et al., 2021). Homostyly formation may also follow long‐distance dispersal and establishment of polyploid (Barrett et al., 2009; Barrett & Shore, 1987; Naiki, 2012). Based on the genetic studies of heterostyly, homostyly can arise from rare crossovers and/or mutations of the *S*‐locus linkage genes (Barrett & Shore, 2008; Charlesworth & Charlesworth, 1979; Ernst, 1955; Li et al., 2016; Yuan et al., 2019), or unlinked modifier genes that have small effects and are not linked with *S*‐locus (Ganders, 1979; Mather & de Winton, 1941). The homostyly formed by modifiers is characterized by many phenotypic variations in the positions of stigmas and anthers (Brys & Jacquemyn, 2015). The patterns of phenotypic variation in heterostylous populations have important implications for the origin pathway of the homostyly (Yuan et al., 2017), although the accurate conclusion depends on further genetic analysis.

Consistent with the shift from outcrossing to autonomous self‐fertilization in monomorphic species, the transition to homostyly in heterostylous groups is accompanied by remarkable floral morphology and metabolite changes. In these autogamous plants, the flowers have a reduced corolla, nectar and pollen yield, low floral scent emission, and a lost herkogamy (stigma‐anther separation) (de Vos et al., 2014; Sicard & Lenhard, 2011; Wu et al., 2017; Zeng et al., 2022; Zhong et al., 2019). The floral scent is a key ecological adaptation signal for interacting plants and pollinators (Farré‐Armengol et al., 2013; Majetic et al., 2009). More than 1700 floral scent components exist, including terpenoids, benzenoids, and fatty acid derivatives (Knudsen et al., 2006). These promote outcrossing and propagation of flowers by luring insect pollinators and will reduce emission and/or change chemical composition after a transition from outcrossing to selfing (Doubleday et al., 2013; Majetic et al., 2019; Petrén et al., 2021; Sas et al., 2016). Although considerable studies have been done on the transition of mating system in monomorphic groups, few studies have focused on the changes of floral scent in the shift from heterostyly to homostyly (Zeng et al., 2022).

**FIGURE 1:**
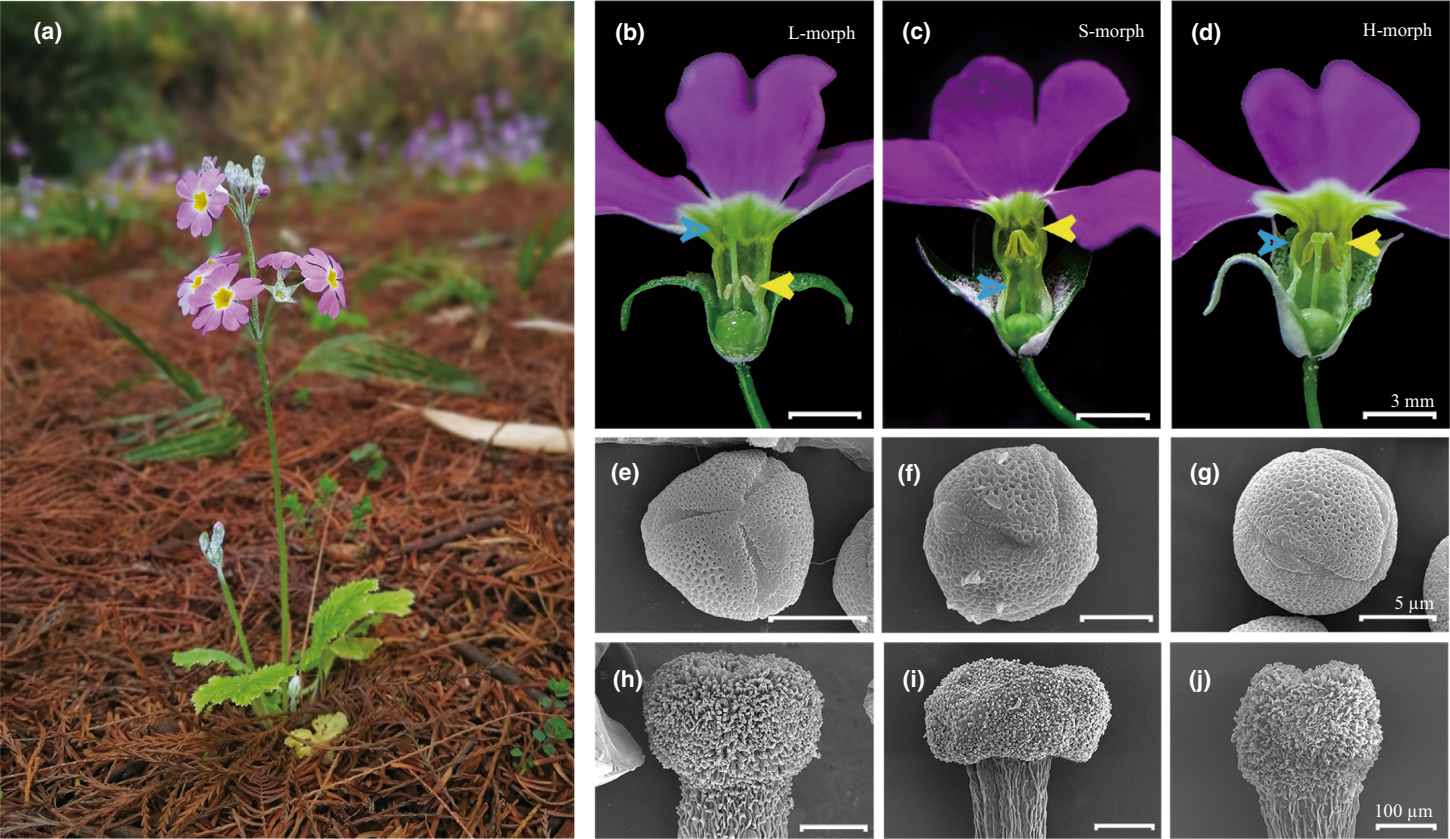
Flowers and morphology of pollen and stigma of *Primula forbesii*. (a) *P. forbesii* flowering plants of HLT population. (b–d) Dissected L‐, S‐, and H‐morph flowers, respectively, showing differences in style length (Blue arrowheads) and anther position (yellow arrowheads). (e–g) Characteristics of pollen grains from L‐, S‐ and H‐morph flowers, respectively, under a scanning electron microscope. (h–j) Morphology of stigmas from L‐, S‐, and H‐morph flowers, respectively.

*Primula* (Primulaceae) is a well‐known distylous genus, with a lot of concerns for more than a century since Darwin's pioneering research (Darwin, 1877). The majority of the 430 species are distylous (95%), while the remaining are homostylous (de Vos et al., 2014; Mast et al., 2006; Richards, 2003). Phylogenetic analysis of *Primula* indicates a single independent origin of distyly and multiple disruptions to homostyles in the genus (Mast et al., 2006). Intraspecific observations of some *Primula* populations further indicate that homostyly evolved from distylous plants (Brys & Jacquemyn, 2015; Zhou et al., 2017). *Primula forbesii* Franch. (Figure 1a) is a less‐known distylous taxon with a strong self‐incompatibility and a pleasant fragrance (Chen & Hu, 1990; Huu et al., 2022). However, preliminary observations indicate that after about 20 years of greenhouse cultivation, the distylous population of *P. forbesii* now varies in floral morphs, producing homostylous plants with long styles and high stamens (Figure 1b–d). Although the appearance of homostylous plants in distyled populations is common, it has been rarely reported that the mating system transition can occur rapidly in just about 20 years. We, therefore, firstly returned to wild populations where the *P. forbesii* seeds were collected originally and documented the floral morph frequencies and floral morphologies to determine whether the homostyly also appeared. Furthermore, we determined the effects of changes from distyly to homostyly on floral morphologies, self‐incompatibility, floral scent, and pollinator visitation to test whether the transition to selfing reduces investment in characteristics of flower display.

Specifically, we aimed to determine: (1) Do the transitions from distyly to homostyly occur rapidly in about 20 generations of artificial domestication, and what is the selective force resulting in the transition to homostyly? We predict the scarcity of long‐tongued pollinators as the main factor for the occurrence of homostylous plants in the distylous populations. (2) Is there evidence that homostylous variants arise by mutation of the *S*‐locus linkage group controlling distylous syndrome rather than modifier genes? Homostyly produced by mutation possesses stigma papillae and pollen size similar to L‐ and S‐morph, respectively. (3) Have the floral morphology, self‐incompatibility, and floral scent and insects visiting changed in homostylous compared with distylous plants? Previous studies demonstrated that variations of mating system and floral signals could rapidly occur in just 11 generations under pollinator‐mediated selection pressure in monomorphic species (Gervasi & Schiestl, 2017).

## 2 MATERIALS AND METHODS

### 2.1 Plant species and study site

*Primula forbesii* sect. Monocarpicae is a biennial herb (Figure 1a) native to China, mainly distributed in the Yunnan and Sichuan provinces (Chen & Hu, 1990; Zhang et al., 2017). A rosette of *P. forbesii* produces 15–22 inflorescences, and each scape bears 15–20 rose‐purple flowers. *P. forbesii* blooms from December to April, and one flower withers 20–24 days after anthesis. The capsules mature between March and May, splitting and releasing tiny seeds (thousand seed weight: 114.40 ± 2.53 mg).

In the spring of 2000, the full‐grown *P. forbesii* plants were collected from two wild distylous populations at Heilongtan (102°45′14″E, 25°8′38″N, HLT) and Xishan (102°37′34″E, 24°58′24″N, XS) in Yunnan province, China. The plants were then mixed and cultivated in the glasshouse at Sichuan Agricultural University, Chengdu Campus (103°51′44″E, 30°42′17″N, CD). The open‐pollinated seeds were randomly collected and sown every year. During the whole domestication process, we did not carry out directional breeding. However, we noticed the variants of homostyly for the first time in 2018. In the spring of 2020–2022, we returned to HLT and XS to investigate whether homostylous plants had appeared in wild populations of *P. forbesii*‐like cultivated populations. Meanwhile, the seeds of cultivated *P. forbesii*, which were randomly collected in the spring of 2019, were sown in the seedling tray in July 2020 in the greenhouse at Sichuan Agricultural University. After 1 month, more than 3000 seedlings were transplanted into 10 × 15 cm plastic pots containing peat and soil and watered every 3 days until flowering.

### 2.2 Floral morph frequency and morphological characteristics

We conducted field investigations in the spring of 2020 and 2021 recorded floral morphs in 316 HLT and 389 XS *P. forbesii* flowering individuals. For the cultivated population CD, we randomly set up 10 quadrats of 10 × 10 pots, each at an interval of 1–2 m, and recorded floral morphs. To sample randomly, we did not replace plants that had not bloomed or died in the quadrats. Therefore, the effective sample size of population CD is 936 plants. We used *χ* ^2^‐tests using SPSS version 23.0 (IBM Corp., Armonk, NY, USA) to determine whether the frequencies of L‐ and S‐morph in cultivated and wild populations had deviated from the expected balanced ratio of 1:1.

For the floral morphological traits, the newly opened flowers, including 40 of each L‐, S‐, and H‐morphs from HLT, XS, and CD populations, were collected and slit longitudinally. The stigma and anther height, herkogamy (stigma‐anther separation), length and width of the corolla tube, and corolla diameter were measured using a digital calliper with an accuracy of 0.01 mm (Brys & Jacquemyn, 2015). About 10–15 buds were collected from 10 plants of each floral morph of three populations to count pollen grains and ovules. The anthers were separated and dried in an oven at 40°C for 12 h, then mixed with distilled water in a 2 ml calibrated tube. The suspension was shaken for 30s, and three independent samples of 1 μl were separately used for slide preparation. The pollen grains were observed and photographed under an optical microscope and counted using ImageJ software (Wu et al., 2015). The ovaries were carefully dissected, and ovules counted under a stereomicroscope (Shao et al., 2019). We randomly collected 10 flowers from each floral morph to measure nectar. The nectar was collected using 7 μl micro‐capillary tubes at 8:00–11:00 am after flowering. A portable refractometer (Brix 0%–80%; CJM‐32, China) was then used to measure the total sugar content in each sample with seven replicates.

The shape, size, and exine sculpture of the pollen grains among different populations and floral morphs were determined in anthers collected during early anthesis from five plants. Dissected anthers were dried in an oven at 40°C for 12 h, followed by the scattering of pollen grains on copper stubs with conductive tapes and then observed under a scanning electron microscope (SU3500; Hitachi High‐Tech, Ibaraki, Japan) with each sample replicated three times. Pollen polar‐ and equatorial axis were measured using ImageJ. Three styles from three plants were collected to determine the variation of stigma papillae cells in L‐, S‐, and H‐morph plants. The styles were placed in glutaraldehyde and osmium tetroxide and then dehydrated for 15 min with ethanol in a series beginning with 30%, followed by 50%, 60%, 70%, 80%, 90%, 95%, and 100%; (Massinga et al., 2005). The characteristics of stigma were observed using a scanning electron microscope (EM CPD300; Leica; Germany) after critical point drying. We used a one‐way analysis of variance (ANOVA) to test the significant differences in floral morphological and ancillary traits between cultivated and wild populations and a *t*‐test to determine the considerable variations between homostyly and distyly in SPSS version 23.0.

### 2.3 Pollen viability and longevity

To compare pollen viability and longevity in different flower morphs, pollen grains from five plants of each floral morph in the cultivated population were collected on the 0, 2, 4, 6, 10, and 14th day after anthesis, respectively. The pollen was cultured in a medium containing 15% sucrose, 2 × 10^−3^ M H_3_BO_3_ and 2 × 10^−3^ M Ca (NO_3_)_2_, pH 5.6, in 25°C, and 90% humidity for 4h. The pollen grain was considered germinated when the pollen tube exceeded its diameter. More than 100 pollen grains were counted in every five repetitions to confirm the pollen germination rate *in vitro*. The significant differences in pollen viability among three floral morphs at different time intervals were determined based on a generalized linear model (GLM).

### 2.4 Variation of self‐incompatibility

We conducted artificial pollination in a glasshouse to confirm the pollen tube growth rate and self‐incompatibility intensity of L‐, S‐, and H‐morphs in the cultivated population of *P. forbesii*. The treatments included: (1) intermorph, intramorph, and selfing pollinations in the L‐ and S‐morphs; and (2) intramorph and selfing pollinations in the H‐morphs. To determine the pollen tube growth rate, each pollination type included 35 flowers from at least seven plants with five flowers fixed in 50% FAA for 12h per time interval (4, 8, 12, 24, 48, 96, and 144 h). After washing three times with distilled water, the styles were cleared in 8 mol L^−1^ NaOH solution at 25°C for 20 min, then rinsed three times again with distilled water before dying with 0.1% aniline blue for 25 min (Lu et al., 2018). More than 10 pollen tubes of each flower were then measured in transparent pistil tissue by fluorescence microscope (BX53‐DP80; Olympus).

To elucidate the self‐incompatibility intensity of L‐, H‐, and S‐morph, 12–17 plants with about 150–250 flowers were artificially pollinated for each pollination type, using flowers from the second and third day after anthesis. Pollination plants were caged before and after artificial pollinations to exclude insect visitors from causing outcrossing, while inter‐ and intra‐morph pollination flowers were emasculated before anthesis. For outcrossing pollinations, pollen was collected from more than three flowering plants and blended before deposition. Pollination experiments were carried out in February 2021 and the fruits matured in late March, after which fruit set and seed production per fruit were recorded. The GLM was used to compare fruit set and seed production per fruit among treatments for each floral morph in SPSS version 23.0.

### 2.5 Analysis of floral scent compounds

Newly opened flowers from L‐, S‐, and H‐morph of cultivated population of *P. forbesii* were collected between 7:00 am and 9:00 am during the blooming stage. We randomly took 0.6 g (approximately 15) flower samples from at least 10 plants of each flower morph with five replicates each. Flowers were introduced into 20 ml sterilized clear glass vials and analyzed within 10 min.

The headspace solid‐phase microextraction (HS‐SPME) and chromatography analyses were performed on a gas chromatography–mass spectrometry (GS‐MS) (TQ8050, Shimadzu, Japan) with an automatic sample manager system. Before headspace collection, the SPME (1.1 mm, CARBON‐WR/PDMS) fiber head was thermally conditioned for 6 min at 250°C. Flowers were incubated for 5 min at 50°C before transferring the SPME fiber to the headspace of the vial for 30 min at 50°C to absorb floral scent compounds. Afterward, the fiber was moved to the injection port for thermally desorbing for 2 min at 250°C.

The chromatographic assay was performed on an InertCap Pure‐WAX column (0.25 mm × 30 m × 0.25 μm). The carrier gas was high‐purity helium, and the split mode was applied at a split ratio of 50:1. The column flow rate was 1.43 ml min^−1^, the linear velocity was 43.3 cm s^−1^, and the purge flow rate was 3 ml min^−1^. In the GC‐MS analyses, a temperature program was set as follows: 50°C held for 5 min with a speed of 10°C per min up to 250°C for 2 min. The total operation time was 27.0 min. Mass spectrometry was done using the triple‐quadrupole mass spectrometer (QQQ‐MS/MS). The program was set as follows: the ionization source was electron ionization (EI), the collision gas was high‐purity argon, and the source temperature was 200°C.

Compounds were determined by comparing the collected fragments with those stored in the NIST libraries. Furthermore, the retention indices for each floral scent compound were obtained by n‐alkane standards and compared with those issued in previous reports. We calculated the relative amounts of each volatile compound by dividing the single peak area by the sum of all peak areas and multiplying by 100%. The floral scent intensity was estimated using the total peak area of the floral sample′s chromatogram.

Because the floral volatile data were not normally distributed, a nonparametric *U*‐test was used to compare the relative amounts of each volatile compound between homostylous and distylous plants. To determine the overall floral scent differences among the three flower morphs, nonmetric multidimensional scaling (NMDS) was carried out using Bray–Curtis similarity indices with 1000 permutations. In addition, a permutational multivariate analysis of variance (PERMANOVA) was also carried out using Bray–Curtis similarity indices with 1000 permutations to examine significant differences in total floral scent profile among the investigated three morphs (Okamoto & Su, 2021).

### 2.6 Insect visitors to flowers

To demonstrate whether different morphs affect floral visitation under the same conditions, we established three *P. forbesii* foraging arrays using potted plants in an open greenhouse. Each array consisted of 30 plants randomly placed 20 cm apart, with 10 plants of each L‐, H‐, and S‐morphs. The arrays were placed more than 5 m from each other to avoid mutual interference, and equal numbers of flowers in each floral morph were maintained in each array by removing all old, wilting, and additional flowers.

The experiments were conducted daily for 1 week, from 10:00 am to 15:00 pm. We recorded all visiting insects and counted the visiting number for each floral morph in each bout. Visiting insects were photographed, caught, and returned to the specimen room for identification. We calculated visitation frequency in each floral morph by dividing the number of visited flowers by the total amount of flowers in each array. We also compared visitation frequencies of morphs of *P. forbesii* using one‐way ANOVA in SPSS 23.0.

## 3 RESULTS

### 3.1 Floral morph frequency

Homostylous individuals were found in the cultivated population CD, with the L‐morph having a significantly higher frequency than S‐morph (*χ* ^2^ = 4.57, df = 1, *p* = .04; Table 1). However, there was no homostyly in both HLT and XS populations, with the floral morph frequencies not deviating from each other L: S = 1: 1 (HLT: *χ* ^2^ = 0.11, df = 1, *p* = .78; XS: *χ* ^2^ = 0.21, df = 1, *p* = .69).

**TABLE 1:** Imformation of location, elevation and floral morph frequency of CD, HLT, and XS populations of *Primula forbesii*.

Population	Location	Elevation	Latitude (N), Longitude (E)	Sample size	Ratio			*χ* ^2^ value
					L	S	H	
CD	Chengdu	535	30°42′17″, 103°51′44″	936	0.48	0.42	0.10	4.57*
HLT	Heilongtan	1923	25°8′38″, 102°45′14″	316	0.51	0.49	0	0.11
XS	Xishan	2018	24°58′24″, 102°37′34″	389	0.51	0.49	0	0.21

*Note*: L, S, and H represent L‐morph, S‐morph, and H‐morph; all *χ* ^2^ are tested against the deviation from 1L: 1S.

* Indicate significant difference at *p* < .05 among morphs.

### 3.2 Floral morphology and ancillary traits

**FIGURE 2:**
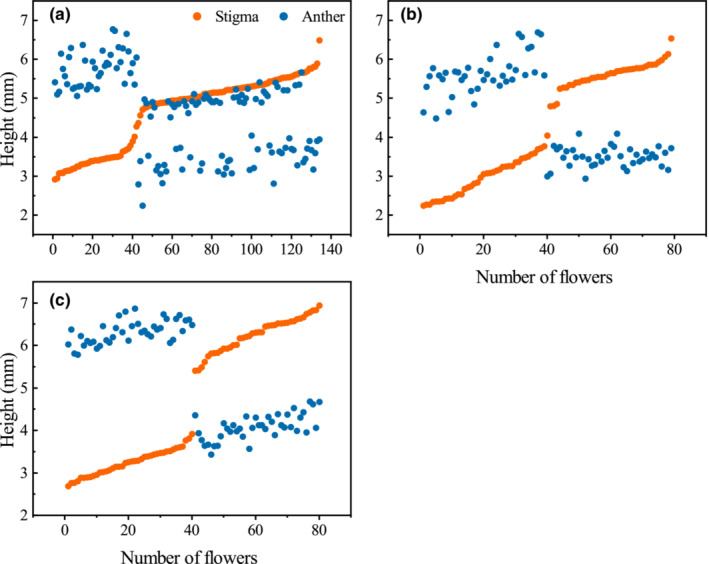
Scatterplots of the height of stigma and anther of each sampled flower at CD (a), HLT (b), and XS (c) populations of *Primula forbesii*.

Flowers of plants growing in a cultivated environment possessed wider and longer floral tubes, larger floral diameters, and higher nectar yield and sugar content than flowers from wild populations (Table 2). However, the herkogamy, pollen–ovule ratio (*P/O*), and the polar and equatorial ratio of pollen (*P/E*) of flowers in cultivated populations reduced significantly relative to wild populations (*p* < .05; Table 2; Appendix S1). The pollen grains of plants grown in cultivated were spherical and spindle‐shaped in wild populations (Figure 1e–g; Appendix S2). In the three populations, the height of stigma and anthers of L‐ and S‐morph individuals displayed a discrete dimorphism, while the homostylous flowers in the cultivated population showed a unimodal distribution (Figure 2).

**TABLE 2:** Differences in floral characteristics and ancillary traits (mean ± SE) among different populations and morphs in *Primula forbesii*.

Floral traits	Population	Mean			*t*‐value	
		L‐morph	S‐morph	H‐morph	H‐morph vs. L‐morph	H‐morph vs. S‐morph
Stigma height (mm)	CD	5.28 ± 0.06c	3.41 ± 0.04a	5.18 ± 0.04	1.55 ns	32.39***
	HLT	5.57 ± 0.07b	2.95 ± 0.08c	–	–	–
	XS	6.25 ± 0.07a	3.25 ± 0.05b	–	–	–
Anther height (mm)	CD	3.40 ± 0.05b	5.78 ± 0.07b	5.01 ± 0.03	−25.33***	9.95***
	HLT	3.50 ± 0.04b	5.63 ± 0.08b	–	–	–
	XS	4.09 ± 0.05a	6.32 ± 0.04a	–	–	–
Herkogamy (mm)	CD	1.88 ± 0.05b	2.37 ± 0.07c	0.19 ± 0.02	30.36***	31.08***
	HLT	2.07 ± 0.07a	2.68 ± 0.07b	–	–	–
	XS	2.17 ± 0.05a	3.08 ± 0.04a	–	–	–
Corolla tube length (mm)	CD	5.43 ± 0.07a	6.46 ± 0.09a	5.79 ± 0.06	−3.85***	6.01***
	HLT	5.41 ± 0.1a	6.14 ± 0.08b	–	–	–
	XS	5.44 ± 0.07a	6.25 ± 0.08ab	–	–	–
Corolla tube width (mm)	CD	2.37 ± 0.05a	3.06 ± 0.03a	2.14 ± 0.04	3.81***	17.74***
	HLT	1.92 ± 0.05b	1.91 ± 0.07b	–	–	–
	XS	1.67 ± 0.04c	2.02 ± 0.07b	–	–	–
Corolla diameter (mm)	CD	19.93 ± 0.27a	20.67 ± 0.26a	18.99 ± 0.33	2.18*	3.92***
	HLT	16.61 ± 0.21c	15.83 ± 0.22c	–	–	–
	XS	18.12 ± 0.23b	17.32 ± 0.15b	–	–	–
*P/O*	CD	1358.09 ± 69.88b	445.8 ± 49.68b	1731.87 ± 129.11	−2.55*	−9.30***
	HLT	1763.88 ± 117.44a	843.52 ± 71.2a	–	–	–
	XS	1826.59 ± 148.95a	943.38 ± 108.38a	–	–	–
Nectar (μl)	CD	0.19 ± 0.01a	0.24 ± 0.02a	0.22 ± 0.02	−1.34 ns	0.78 ns
	HLT	0.11 ± 0.01b	0.11 ± 0.01b	–	–	–
	XS	0.11 ± 0.02b	0.1 ± 0.01b	–	–	–
Sugar content (%)	CD	65.29 ± 1.51a	57.14 ± 4.28a	62.43 ± 1.88	1.19 ns	−1.13 ns
	HLT	50.57 ± 5.25b	49.57 ± 3.81a	–	–	–
	XS	47.86 ± 4.82b	53.71 ± 4.09a	–	–	–

*Note*: The floral traits (Mean ± SE) having different letters within a column indicate significant difference (*p* < .05) among three populations based on one‐way analysis of variance.

Abbreviations: *P/O*, pollen–ovule ratio; “–” indicates no observation was made.

*Significant difference at *p* < .05; **significant difference at *p* < .01; ***significant difference at *p* < .001 based on *t*‐test.

In the cultivated population, the homostyles possessed high‐level stigma (5.18 ± 0.04 mm) and anthers (5.01 ± 0.03 mm) but almost completely lost herkogamy (0.19 ± 0.02 mm) in comparison with L‐ (1.88 ± 0.05 mm) and S‐morphs (2.37 ± 0.07 mm; *p* < .01; Figure 1b–d; Table 2). As expected, the homostyles of *P. forbesii* possessed pollen size close to S‐morphs and stigma papillae near to L‐morphs (Figure 1e–j). The corolla tube length of homostyles was significantly larger than L‐morphs and smaller than S‐morphs (*p* < .01); however, the corolla tube width and corolla diameter were significantly smaller than both the L‐morphs and S‐morphs (*p* < .01). Furthermore, the pollen count of homostyles was significantly more than S‐morphs and less than L‐morph. On the anther, the ovule number per flower of homostyles was substantially less than both L‐ and S‐morphs (*p* < .05; Table 2; Appendix S1). As a result, the *P/O* value of homostyles was significantly higher than that of distylous flowers (*p* < .05). However, there were no significant variations in both the nectar yield and sugar content between homostylous and distylous flowers (*p* > .05).

### 3.3 Pollen viability and longevity

**FIGURE 3:**
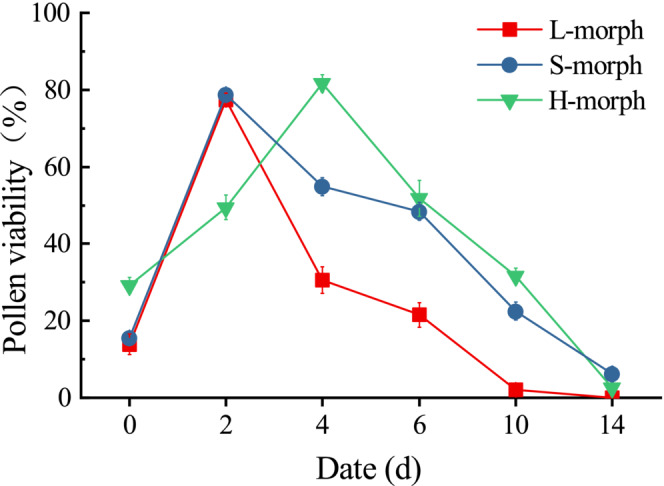
Pollen viability of three floral morphs in *Primula forbesii* determined by germination *in vitro*. Data are shown as means, and the bars indicate the standard error.

The viabilities of L‐ and S‐morph pollens reached their peaks on the second day after flowering (77.34 ± 1.68% and 78.67 ± 1.10%, respectively) and were significantly higher than homostyles (49.48 ± 3.19%; *p* < .001; Figure 3; Appendix S3). However, the pollen viability of homostylous flowers reached its peak on the fourth day after anthesis (81.68 ± 2.28%) and was significantly higher than viability in L‐morphs and S‐morphs (*p* < .001). The viabilities of S‐ and H‐morphs declined more slowly than L‐morph after reaching the peak, with the pollen grains of all three floral morphs losing viability after 14 days of flowering. In addition, the pollen tubes of S‐morph had the fastest growth rate in the standard medium, followed by tubes of H‐ and L‐morph, respectively (Appendix S4). The results demonstrate that the pollens of the three flower morphs reach their maximum viability in 2–4 days after flowering.

### 3.4 Patterns of pollen tube growth and self‐incompatibility status

**FIGURE 4:**
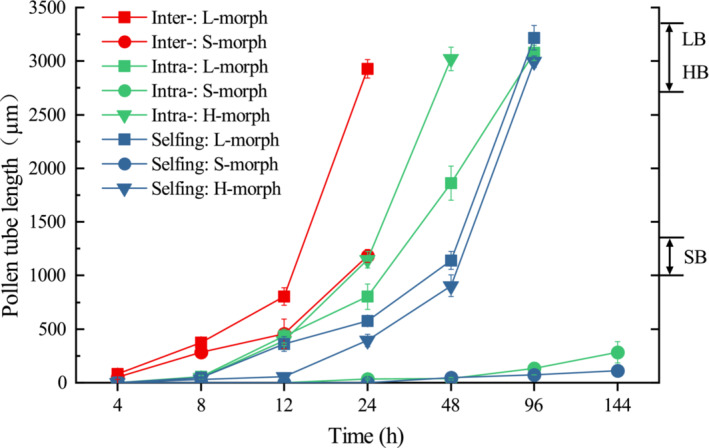
Patterns of pollen tube growth of *Primula forbesii* in different artificial pollination. Data are shown as means, and the bars indicate the standard deviation. Red lines with “Inter” indicate intermorph pollinations; green lines with “Intra” indicate intramorph pollinations; blue lines with “Selfing” indicate Selfing pollination; LB, base of L‐morph styles; SB, base of S‐morph styles; HB, base of H‐morph styles.

**FIGURE 5:**
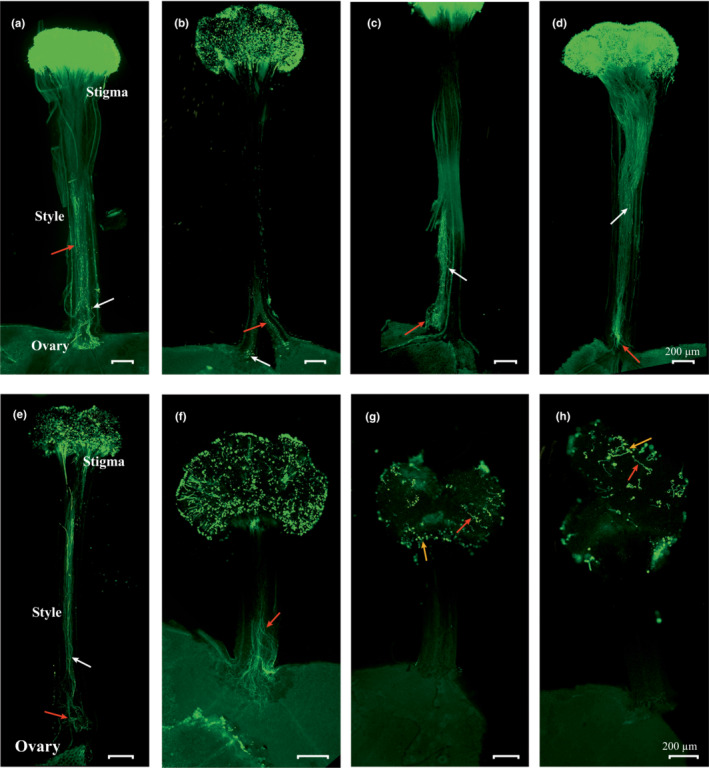
States of pollen tube growth of three floral morphs following intermorph, intra‐morph and self‐crossing in *Primula forbesii*. (a) L‐ × S‐morph pollen tubes arrival at ovary 24 h after pollination; (b,c) intra‐morph and selfed L‐morph pollen tubes arrival at ovary 96 h after pollination; (d,e) intra‐morph and selfed H‐morph pollen tubes arrival at ovary 48 h and 96 h after pollination, respectively; (f) S‐ × L‐morph pollen tubes arrival at the ovary 24 h after pollination; (g,h) a small amount of germination and abnormal growth of intra‐morph and selfed S‐morph pollen grains 144 h after pollination. All scale bars = 200 μm. Red arrows represent the pollen tubes; white arrows represent the callose plugs; yellow arrows represent the pollen grains.

Our study showed that the pollen germinates on the stigma after 4 or 8 h of pollination in L‐morphs and homostyles (Figure 4). We also found out that the pollen tubes arrive at the ovary in L‐morphs after 96 h of intra‐morph and self‐pollination and 24 h after intermorph pollination (Figure 4; Figure 5a–c). The arrival time of pollen tubes at the ovary in H‐morphs was 48 and 96 h of intra‐morph and self‐pollination, respectively (Figure 5d,e). In S‐morphs, the pollen grain germinated on the stigma within 4 h and reached the ovary at the 24th hour after intermorph pollination (Figure 5f). A small number of irregular distorted and swollen pollen tubes also emerged but did not reach the ovary in intra‐morph and self‐pollination (Figure 5g,h).

**FIGURE 6:**
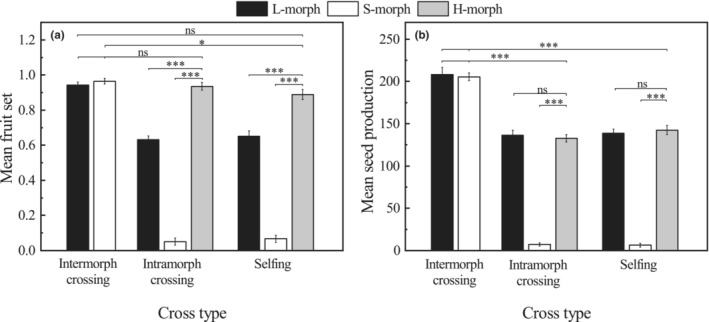
Differences in fruit set (a) and seed production per fruit (b) of *Primula forbesii* after controlled pollination in intermorph, intra‐morph and self‐pollinated populations. *Significant differences at *p* < .05 or very significant at **p < .01; ***p < .001 based on the generalize linear model. Data are shown as means, and the bars indicate the standard errors.

The study revealed that the distylous plants in the intermorph pollination had the largest fruit set and seed production per fruit compared with all other pollination combinations (Figure 6). The fruit sets of homostyles in intra‐morph (93.56 ± 2.26%) and self‐pollination (88.86 ± 2.83%) were significantly higher than those recorded in L‐ and S‐morphs (*p* < .05) and close to distylous plants of intermorph pollinations (Figure 6a). The seed production per fruit of homostyles in intra‐morph (132.75 ± 4.30) and selfing (142.5 ± 5.34) was similar to those produced in L‐morphs (*p* > .05) but significantly higher than those in S‐morph (*p* < .05). In addition, they were all significantly lower than that of distylous plants of intermorph pollination (*p* < .05; Figure 6b). Under illegitimate pollination, the L‐morphs showed partial fitness, with about 60% fruit set, while the S‐morphs showed completely heteromorphic incompatibility, with no seed production.

### 3.5 Analysis of floral scent compounds

A total of 74 floral volatiles were detected, belonging to three classes of compounds, with 15 terpenoids, 32 benzenoids, and 27 fatty acid derivatives. The predominant floral scent compounds of *P. forbesii* were linalool, cis‐3‐hexen‐1‐ol, benzeneacetonitrile, benzyl alcohol, and alpha‐terpineol (Appendices S5 and S6).

**FIGURE 7:**
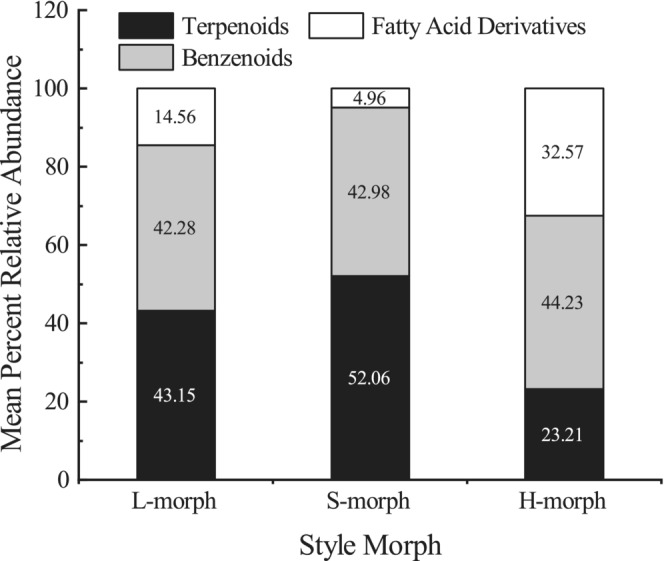
Mean percent relative abundance of floral scent compound classes for L‐, S‐, and H‐morphs of *Primula forbesii*.

The relative amount of terpenoids found in homostyles was significantly lower than in distylous flowers (*p <* .01), unlike the fatty acid derivatives, which were significantly higher in homostyles (*p <* .01; Table 3; Figure 7). The benzenoids did not differ considerably between homostyles and distylous flowers (*p >* .01). However, there were variations in some major floral scents (>1%) between homostyles and distylous individuals (Table 3). For example, the amounts of linalool and alpha‐terpineol were significantly higher in distylous flowers than in homostyles (*p <* .01). We also found substantially more cis‐3‐hexen‐1‐ol in homostyles than in distylous flowers (*p <* .01). We used the total peak area to represent floral scent intensity and found out that the concentration of floral volatile in S‐morphs was significantly higher than in L‐ and H‐morph flowers (*p <* .01).

**TABLE 3:** Major (>1%) floral scent compounds (mean ± SE) in three flower morphs of *Primula forbesii*.

No.	Compound	R.T.	R.I.	Mean			*U* value	
				L‐morph (*N* = 5)	S‐morph (*N* = 5)	H‐morph (*N* = 5)	H‐morph vs. L‐morph	H‐morph vs. S‐morph
	Terpenoids			43.15 ± 1.61	52.06 ± 1.92	23.21 ± 0.93	0.00**	0.00**
1	α‐Pinene	3.722	1017	1.09 ± 0.10	2.86 ± 0.29	0.51 ± 0.22	4.00 ns	0.00**
2	β‐Pinene	5.536	1102	1.17 ± 0.26	3.96 ± 0.74	0.46 ± 0.11	3.00*	0.00**
4	1,8‐Cineole	7.663	1190	1.87 ± 0.71	1.77 ± 0.25	1.28 ± 0.56	10.00 ns	10.00 ns
5	Ocimene	8.477	1226	1.01 ± 0.31	1.79 ± 0.42	1.14 ± 0.62	9.00 ns	7.00 ns
6	Linalool	13.878	1539	18.19 ± 2.00	13.23 ± 0.93	7.66 ± 0.75	0.00**	0.00**
7	beta‐elemene	14.467	1580	2.35 ± 0.79	3.09 ± 0.52	2.00 ± 0.36	11.00 ns	6.00 ns
9	(‐)‐alpha‐Gurjunene	15.47	1665	1.90 ± 0.33	4.45 ± 1.72	0.65 ± 0.10	0.00**	0.00**
10	alpha. – Terpineol	15.873	1686	5.91 ± 0.81	9.33 ± 0.67	2.11 ± 0.33	0.00**	0.00**
11	β‐Selinene	15.995	1696	5.71 ± 0.70	7.45 ± 0.30	4.28 ± 0.60	5.00 ns	0.00**
12	(+)‐valencene	16.15	1708	1.31 ± 0.29	2.23 ± 0.23	0.89 ± 0.15	7.00 ns	0.00**
14	Nerolidol	19.852	2027	0.55 ± 0.24	1.64 ± 0.33	1.07 ± 0.40	7.00 ns	5.00 ns
15	Perhydrofarnesyl Acetone	20.811	2117	1.91 ± 0.78	1.03 ± 0.19	1.12 ± 0.35	8.00 ns	12.00 ns
	Benzenoids			42.28 ± 2.41	42.98 ± 1.99	44.23 ± 1.32	10.00 ns	8.00 ns
23	4‐Methylanisole	12.166	1425	4.48 ± 2.16	6.98 ± 1.24	3.77 ± 1.03	12.00 ns	4.00 ns
24	Benzaldehyde	13.486	1512	3.04 ± 0.30	2.95 ± 0.46	4.02 ± 0.53	4.00 ns	4.00 ns
25	Methyl benzoate	14.886	1611	0.98 ± 0.14	0.37 ± 0.03	1.75 ± 0.26	3.00*	0.00**
26	Phenylacetaldehyde	15.158	1632	1.36 ± 0.43	0.22 ± 0.07	1.56 ± 0.35	10.00 ns	0.00**
27	Acetophenone	15.263	1640	1.55 ± 0.28	2.69 ± 0.60	2.16 ± 0.18	4.00 ns	9.00 ns
29	Benzyl Acetate	16.273	1718	1.52 ± 0.26	2.02 ± 0.13	1.53 ± 0.14	12.00 ns	2.00*
31	Methyl salicylate	16.89	1768	6.01 ± 1.84	1.86 ± 0.30	5.14 ± 0.73	11.00 ns	1.00*
34	2‐Methoxyphenol	17.821	1846	1.14 ± 0.27	0.55 ± 0.11	1.54 ± 0.41	10.00 ns	2.00*
35	Benzyl alcohol	17.992	1860	7.07 ± 1.29	9.51 ± 1.13	8.93 ± 1.20	8.00 ns	11.00 ns
36	Phenethyl alcohol	18.408	1896	3.12 ± 0.65	2.83 ± 1.13	2.19 ± 0.61	7.00 ns	12.00 ns
38	Benzeneacetonitrile	18.611	1914	9.34 ± 1.65	10.22 ± 1.48	9.20 ± 0.91	12.00 ns	12.00 ns
	Fatty acid derivatives			14.56 ± 2.61	4.96 ± 0.26	32.57 ± 2.01	0.00**	0.00**
49	cis‐3‐Hexenal	6.328	1135	1.03 ± 0.17	0.21 ± 0.05	1.75 ± 0.42	5.00 ns	0.00**
50	1‐Penten‐3‐ol	7.283	1173	1.01 ± 0.44	0.80 ± 0.10	1.52 ± 0.47	8.00 ns	6.00 ns
51	trans‐2‐Hexenal	8.066	1206	3.08 ± 0.85	0.57 ± 0.14	7.66 ± 1.83	3.00*	0.00**
52	Tetramethylene sulfone	8.203	1215	0.68 ± 0.30	0.48 ± 0.09	2.62 ± 0.70	4.00 ns	5.00**
55	cis‐2‐Penten‐1‐ol	10.303	1318	1.25 ± 0.42	0.50 ± 0.13	2.57 ± 0.61	4.00 ns	0.00**
56	1‐Hexanol	10.872	1350	0.89 ± 0.35	0.25 ± 0.03	1.28 ± 0.28	9.00 ns	0.00**
57	cis‐3‐Hexen‐1‐ol	11.394	1380	5.17 ± 1.23	1.43 ± 0.10	13.72 ± 1.52	0.00**	0.00**
	Total Peak Area (billion)			2.66 ± 0.19	4.57 ± 0.09	3.12 ± 0.27	6.00 ns	0.00**

Abbreviations: R.T., retention time; R.I., Retention index.

*Significant difference at *p* < .05 between morphs or **very significant at *p* < .01 based on Mann–Whitney *U*‐tests.

**FIGURE 8:**
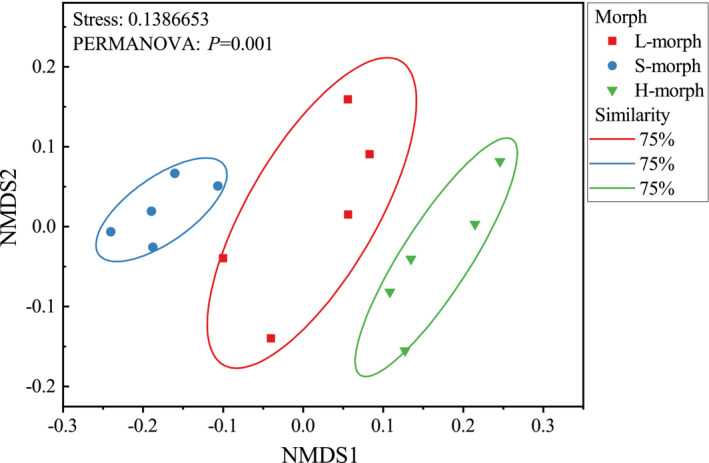
Non‐metric multi‐dimensional scaling (NMDS) analyses of floral scent compositions of L‐, S‐, and H‐morph flowers in *Primula forbesii*. The 2D stress value is 0.1386653. Difference was tested based on permutational multivariate analysis of variance (PERMANOVA).

The NMDS plot displayed a clear separation among the three floral morphs based on their floral scent composition (Figure 8). This is supported by PERMANOVA analysis, which also revealed significant differences in floral scent profile among floral morphs (*F* = 4.54, df = 2, *p* = .001). Furthermore, the floral scent profile of homostyles was significantly divergent from S‐morphs (*F* = 50.62, df = 1, *R* ^2^ = .86, *p* = .01) but similar to L‐morphs (*F* = 1.85, df = 1, *R* ^2^ = .19, *p* = .20; Table 4). Apparent divergence also exists in the floral scent composition between L‐ and S‐morphs (*F* = 19.96, df = 1, *R* ^2^ = .71, *p* = .01).

**TABLE 4:** Results of PERMANOVA for floral scents emitted from flowers of L‐, S‐, and H‐morphs of *Primula forbesii*.

Pairs	df	*F*	*R* ^2^	*p*
L‐morph vs. H‐morph	1	1.850674749	.187872892	.195 ns
L‐morph vs. S‐morph	1	50.62428116	.863537772	.01*
H‐morph vs. S‐morph	1	19.95748785	.713851257	.009**

*Significant difference at *p* < .05 between morphs or **very significant at *p* < .01 based on *t*‐test.

### 3.6 Insect visitation to flowers

**FIGURE 9:**
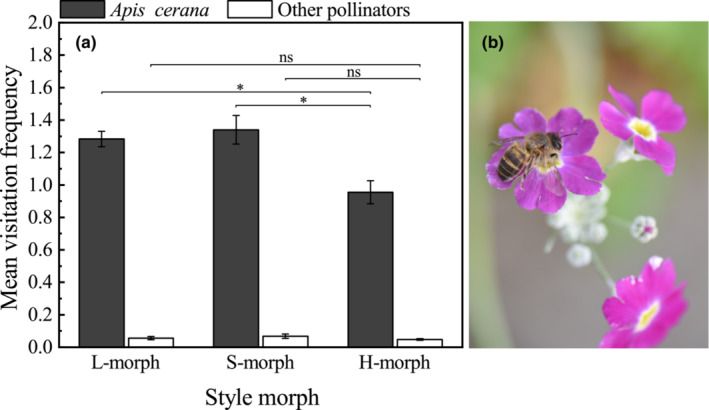
Mean (±SE) visitation frequencies of the pollinator insects on different floral morphs of *Primula forbesii* (a) and the main pollinator *Apis cerana* Fabricius (b). *Significant differences at *p* < .05 based on one‐way ANOVA. Vertical bars indicate the standard errors.

Most *P. forbesii* pollinators observed in the open greenhouse were short‐tongued honeybees of the species *Apis cerana* Fabricius (Figure 9). Only a small number of other pollinators appeared, including some bumblebees and butterflies. Because of their low numbers, they had little impact on the propagation of *P. forbesii*. According to visitation frequencies, *Apis cerana* significantly preferred flowers on L‐ (1.28 ± 0.05) and S‐morphs (1.34 ± 0.08) over H‐morph flowers (0.95 ± 0.07; *p* < .05). For other pollinators, there were no differences in visitation frequencies (L‐morph 0.06 ± 0.01, H‐morph 0.05 ± 0.01, S‐morph 0.07 ± 0.01).

## 4 DISCUSSION

Our analysis of the transitions from outcrossing to selfing in *P. forbesii* obtained several key findings. We showed that the unreliable short‐tongued *A*. *cerana* might be the main selective force for the variations from distyly to homostyly in a cultivated population of *P. forbesii*. The comparison between cultivated populations and ancestral wild populations also indicated that alterations in the mating system occurred rapidly in just about 20 years of domestication. Moreover, homostylous *P. forbesii* possessed stigma papillae similar to L‐morph and anthers similar to S‐morph, indicating that the variants were long homostyly, which possibly originated from crossovers and mutations at *S‐* linkage region governing the distylous syndrome, although polygenic modifiers could also be have been involved. The study also found that the shift from outcrossing to selfing was accompanied by reduced flower attraction, especially the smaller corolla diameter, reduced floral scent emission, changed floral scent composition, and lower visitation compared with the distylous plants. Our work demonstrates that the changes in the mating system and floral traits may occur faster with the changes in the growth environment, especially in changed pollinator communities, which will provide new insights into the ecological and evolutionary studies of heterostyly.

### 4.1 Ecological pressure of homostyly evolution

Compared with the original habitats, the artificial cultivation or city environment may be characterized by increased spatial fragmentation and depauperate pollinator fauna, which would make the populations be affected by demographics, environment, and genetic stochasticity (Barmentlo et al., 2018; Oostermeijer et al., 2003). When unreliable pollinators limit outcrossing seed sets, these unfavorable environments might favor variations in autonomous self‐pollination for the production and spread of obligate outcrossing population, which is the Darwin's reproductive assurance hypothesis (Busch & Delph, 2012; Darwin, 1876; Moeller, 2006). The hypothesis is supported by the studies of numerous *Primula* species, in which self‐incompatible distyly frequently breaks down into self‐compatible homostyly (Carlson et al., 2008; Piper et al., 1986; Richards, 2003; Yuan et al., 2017). Based on higher herkogamy and the isoplethic equilibrium of L1:S1, outcrossing is more prevalent in wild populations of *P. forbesii* than the cultivated population. On the contrary, the cultivated population was biased to the partially self‐compatible L‐morph and produced the completely self‐compatible homostyly. We found that the main pollinators of the cultivated populations were the short‐tongued *A. cerana* (about 5 mm), and the occasional visits by long‐tongued bumblebees and butterflies have little contribution to this species. Because long‐tongued pollinators mediate intermorph pollen transfer by contacting both the high‐ and low‐level sex organs, they are considered more effective pollinators in distylous species with tubular flowers (Lloyd & Webb, 1992; Santos‐Gally et al., 2013; Yuan et al., 2017). The corolla tube length of *P. forbesii* ranges from 5.43 to 6.67 mm, making it difficult for *A. cerana* to mediate disassortative pollen transfer between the anthers of L‐morph and stigma of S‐morph. Furthermore, the natural selection pressure due to the variable pollinator communities can rapidly alter the floral traits and mating system (Gervasi & Schiestl, 2017). Therefore, limited pollen of crossed seed sets caused by unreliable pollinators may be the main selective force for the fast variations of the mating system in *P. forbesii* in only 20 years. Nevertheless, more open pollination experiments are necessary to determine the productive pressure between distyly and homostyly.

Some variations in morphological and ancillary traits between wild and cultivated populations might be attributed to environmental conditions. For example, plants in the cultivated population had larger flowers with more nectar. The sufficient nutrient supply and stable growing condition of the artificial environment might be the main reasons for these differences, which are also supported by the significantly increased plant height and crown in the cultivated population (data not shown). In addition, the variation in pollen grain morphology among different populations is consistent with variations in previous studies (Li & Johnston, 2001; Xu et al., 2019). Morozowska and Idzikowska (2004) found significant differences in pollen morphologies between natural and cultivated populations in *P. veris*, which may be the result of adaptive change or/and random mutation due to geographical isolation.

### 4.2 Origination of homostyly

Homostyly originates from the rare crossovers or/and mutations of *S*‐locus linkage genes governing distylous syndrome. In contrast with the gradual evolution of self‐pollination characteristic of most angiosperm groups, the variations of the mating system in distylous species could arise rapidly in a few generations (Kappel et al., 2017). The long homostyly, which is the most common type, combines the female organ of L‐morph with long papillae cells and the male organ of S‐morph with large pollen grains and is usually associated with the breakdown of heteromorphic self‐incompatibility and a high capability of autonomous self‐fertilization (Barrett, 1992; Barrett & Shore, 2008; Haddadchi & Fatemi, 2015; Li & Johnston, 2001). The vast majority of homostyly in *Primula* species arose from this pathway (Ernst, 1955; Xu et al., 2019; Yuan et al., 2017; Zhou et al., 2017). Recent studies of genetic architecture reveal that the *S‐*linkage group governing *Primula* distylous syndrome is a hemizygous region of several genes that is only present in S‐morph (*S‐*) and absent in L‐morph (*‐ ‐*) (Li et al., 2016). This finding indicates that the long homostyly more likely originated from mutations of large effect genes in the *S* region rather than homologous recombination. Another alternative pathway of homostyly origin involved the modifier genes unlinked with *S*‐locus. Through this approach, long homostyly might arise directly from the variations of the stamen of L‐morph or pistil of S‐morph, and it possesses pollen size, stigma papillae, and self‐incompatibility similar to L‐ or S‐morphs (Brys & Jacquemyn, 2015; Ganders, 1979; Mather & de Winton, 1941). Additionally, the long homostyly caused by polygenic modifiers would present strong variations of sexual organ height (Zhang et al., 2021).

Unfortunately, our data did not determine which evolutionary pathway the homostyly of *P. forbesii* originates from but supported more mutations of the S‐locus linkage group. Based on similar stigma papillae and pollen size to L‐ and S‐morph, respectively, long homostyly more likely arose by mutation of *S*‐locus genes. However, based on similar pollen count to L‐morph, the evolutionary pathway of polygenic modifiers was more likely. Further hybrid experiments and genetic analysis were necessary. However, based on the clear bimodal distribution of anther‐stigma height of the distylous plants, the long homostyly probably originated from the mutations of large effect genes in S‐locus region, although modifier genes might also contribute to several variations in homostylous syndrome, as predicted by Zhou et al. (2017).

### 4.3 Evolutionary consequences of homostyly

Comparisons of the ecologically functional characteristics between distyly and homostyly can reveal the consequences of pollination and reproductive biology on the variations of the mating system (Yuan et al., 2017). In our study, the flowers of *P. forbesii* distylous plants were polymorphic with reciprocal stigma‐anther position and dimorphic stigma and pollen. However, the homostyles lost herkogamy. Reduced or lost herkogamy indicates mating system transitions by providing opportunities for autonomous self‐pollination (Barrett & Shore, 1987; de Vos et al., 2012; Ganders et al., 1985; Takebayashi et al., 2006; Wu et al., 2017; Yuan et al., 2017). Additionally, the transitions from distyly to homostyly in *P. forbesii* were also associated with reduced corolla diameter, similar to other *Primula* species (Li & Johnston, 2001; Zhong et al., 2019; Zhou et al., 2017). Since pollinators are no longer necessary, the floral traits associated with pollination can be reduced through relaxed selection or genetic drift, leading to selfing syndrome (Barrett et al., 2009; Goodwillie et al., 2010; Sicard & Lenhard, 2011). However, selfing syndrome is not inevitable in the shift from outcrossing to selfing but depends on the history, advantages, and intensity of selfing and the opportunities of outcrossing in populations (de Vos et al., 2014). This explains the increased pollen count and *P/O* value in homostyles compared with S‐morph plants in this study.

In many *Primula* species, S‐morph has stricter heteromorphic self‐incompatibility than L‐morph (Chen, 2009; Huang et al., 2015; Jiang & Li, 2017; Wedderburn & Richards, 1990). This is consistent with our results in which the L‐morphs were partially self‐compatible, and S‐morphs were completely self‐incompatible. Moreover, the illegitimate pollen tube on L‐morph styles partially entered the ovary but could not germinate on the S‐morph stigma. The performances of incompatible pollen indicate that the female incompatibility of S‐morph is entirely sporophytic but is gametophytic in L‐morph (Lewis & Jones, 1992; McCubbin, 2008). Recently, Huu et al. (2022) demonstrated that concentrations of brassinosteroids during the development of female organs of *P. forbesii* probably involve style elongation and the formation of female self‐incompatibility, which provides new insights into heteromorphic self‐incompatibility study. The high selfing rate of homostyly is widely reported in distylous groups (Barrett, 2019; Barrett & Shore, 2008; Darwin, 1877; Ganders, 1979; Ganders et al., 1985). Compared with distylous individuals of *P. forbesii*, the homostyles were completely self‐compatible with 90% fruit set. Although the seed production due to self‐pollination of homostyly was significantly lower than intermorph pollination of distyly, self‐fertilized homostyles were preferred in self‐incompatible distylous population when unreliable short‐tongued *A. cerana* pollinators limited the effectively intermorph pollen transfer between L‐ and S‐morph.

The floral scent promotes reproductive success by attracting pollination insects (Majetic et al., 2009). Previous studies found that floral scent varied significantly among different *Primula* species, but not between the L‐ and S‐morphs of each species (Gaskett et al., 2005; Zeng et al., 2022). However, similar to Johnson et al. (2019), we found significant differences in the intensity and composition of floral scent between L‐ and the S‐morphs of *P. forbesii*, with reduced floral scent emission and terpenoids content in L‐morph. We speculate that the different self‐incompatibility between L‐ and S‐morph may be the main reason of floral scent divergence (Kariyat et al., 2021). The transitions of mating system commonly accompany variations in floral fragrance in many angiosperm lineages (Ferrari et al., 2006; Doubleday et al., 2013; Kariyat et al., 2021; Petrén et al., 2021;). Zeng et al. (2022) showed that the shift from distyly to homostyly was accompanied by reduced floral scent emission and changed floral scent profile in *P. oreodoxa*. This is consistent with our investigations where the homostyles of *P. forbesii* had reduced floral scent intensity and different floral scent profiles from distylous flowers, especially of S‐morphs. The changes in mating style mediated by pollinator groups maybe the main reason for the floral odour differences (Gervasi & Schiestl, 2017). Furthermore, the relative content of terpenoids, especially linalool, was significantly lower in homostylous than in distylous plants. The terpenoids elicit positive guiding behaviors in honey‐ and bumblebees and exist in most bee‐pollinated angiosperms (Dobson, 2006; Dotterl & Vereecken, 2010; Laloi et al., 2001; Parachnowitsch et al., 2013).

In our study, *A. cerana* prefered distylous plants, especially S‐morphs, compared with homostyles. The low pollinator activity in homostylous *P. oreodoxa* has been reported, although with no differences in elevation and size among populations (Yuan et al., 2017). Carr et al. (2014) demonstrated that the outcrossed progeny of *Mimulus guttatus* significantly had a higher visiting frequency than the self‐fertilized offspring, although they came from the same population. This could be due to pollinators strongly discriminating against inbreds (Carr et al., 2015; Ivey & Carr, 2005). A considerable *P. forbesii* homostyles come directly from self‐pollination, leading to divergence in floral scent profiles and reduced visiting frequency in homostylous plants. Moreover, earlier studies showed that pollinators' preference for outcrossing plants still existed, although the corolla size and pollination reward had been controlled (Carr et al., 2014, 2015; Ivey & Carr, 2005). Therefore, pollinator‐mediated natural selection on floral odor may be stronger than flower morphology or color selection in some species (Byers et al., 2014; Parachnowitsch et al., 2013). Collectively, the variations in the floral scent emission and composition during transitions to homostyly seem likely to be associated with reduced pollinator visiting, but further empirical investigations are necessary to determine the relationship between the floral scent variation and pollinator visiting in *P. forbesii*.

## AUTHOR CONTRIBUTIONS

**Cai‐Lei Liu:** Conceptualization (lead); data curation (lead); formal analysis (lead); investigation (lead); methodology (lead); visualization (lead); writing – original draft (lead). **Yin Jia:** Conceptualization (lead); funding acquisition (lead); project administration (lead); resources (lead); supervision (lead); writing – review and editing (equal). **Yi‐Feng Li:** Data curation (equal); formal analysis (equal); investigation (equal); methodology (equal); visualization (equal). **Yuan‐Fen Xiang:** Data curation (equal); formal analysis (equal); investigation (equal); methodology (equal); visualization (equal). **Yuan‐Zhi Pan:** Conceptualization (equal); funding acquisition (equal); resources (equal); supervision (equal); writing – review and editing (equal). **Qinglin Liu:** Conceptualization (equal); investigation (equal); methodology (equal); resources (equal); supervision (equal); writing – review and editing (equal). **Ke‐Hang Ma:** Data curation (equal); formal analysis (equal); investigation (equal); methodology (equal). **Xian‐Cai Yin:** Conceptualization (equal); data curation (equal); investigation (equal); methodology (equal).

## CONFLICT OF INTEREST

The authors declare that the research was conducted in the absence of any commercial or financial relationships that could be construed as a potential conflict of interest.

## Supporting information


Appendix S1.

## Data Availability

All data needed to evaluate the conclusions in the paper are present in the paper and/ or in the Supporting Information.
