# Supplementary material for: The rapid appearance of homostyly in a cultivated distylous population of Primula forbesii
Source: Ecol Evol. 2022 Nov 18;12(11):e9515. doi: 10.1002/ece3.9515 (PMC9674475; doi:10.1002/ece3.9515)

**Appendices**

**The rapid appearance of homostyly in a cultivated distylous population of *Primula forbesii***

Cai-Lei Liu^1, *^, Yin Jia^1, *, 2^, Yi-Feng Li^1^, Yuan-Fen Xiang^1^, Yuan-Zhi Pan^1^, Qing-Lin Liu^1^, Ke-Hang Ma^1^, and Xian-Cai Yin^1^

| **Appendix S1.** Differences in floral and ancillary characteristics (mean ± SE) among different populations and morphs in *Primula forbesii*. | | | | | | |
| --- | --- | --- | --- | --- | --- | --- |
| Floral traits | Population | Mean (± SE) | | | *t* value | |
|  |  | L-morph | S-morph | H-morph | H-morph vs. L-morph | H-morph vs. S-morph |
| No. of pollen grains  per flower | CD | 343600±14650a | 115200±11837b | 289200±17145.65 | 2.41 * | -8.35 *** |
|  | HLT | 328800±22060a | 150667±10171ab | – | – | – |
|  | XS | 354533±27529a | 166800±15743a | – | – | – |
| No. of ovules per flower | CD | 257.47±10.09a | 269.27±13.17a | 187.5±11.32 | 4.53 *** | 4.74 *** |
|  | HLT | 188.53±6.49b | 183.2±7.67b | – | – | – |
|  | XS | 196.8±7.1b | 182.6±6.26b | – | – | – |
| Polar axis (µm) | CD | 8.84±0.12b | 11.14±0.11c | 10.28±0.18 | -6.99 *** | 3.13 ** |
|  | HLT | 12.96±0.12a | 18.04±0.16b | – | – | – |
|  | XS | 13.20±0.13a | 19.57±0.19a | – | – | – |
| Equatorial axis (µm) | CD | 9.35±0.09a | 12.41±0.15a | 11.16±0.16 | -10.40 *** | 5.15 *** |
|  | HLT | 7.32±0.11b | 10.61±0.09b | – | – | – |
|  | XS | 7.44±0.07b | 10.19±0.12c | – | – | – |
| *P/E* | CD | 0.95±0.02b | 0.90±0.02c | 0.92±0.02 | 0.79 ns | -0.89 ns |
|  | HLT | 1.79±0.03a | 1.71±0.02b | – | – | – |
|  | XS | 1.78±0.02a | 1.93±0.03a | – | – | – |
| The floral traits (Mean ± SE) having different letters within a column indicate significantly different (*P*<0.05) among three populations based on One-way ANOVA; "ns" indicate no significantly different based on *t*-test (*P*>0.05); *P/E*, polar and equatorial ratio of pollen grain; "－"indicate not existed. | | | | | | |

**Appendix S2.** Morphology of pollen grain of wild *Primula forbesii* populations. (A, B) Morphology of pollen grains from L- and S-morph flowers of HLT population under scanning electron microscope. (C, D) Morphology of pollen grains from L- and S-morph flowers of XS population. All bars = 5 µm.


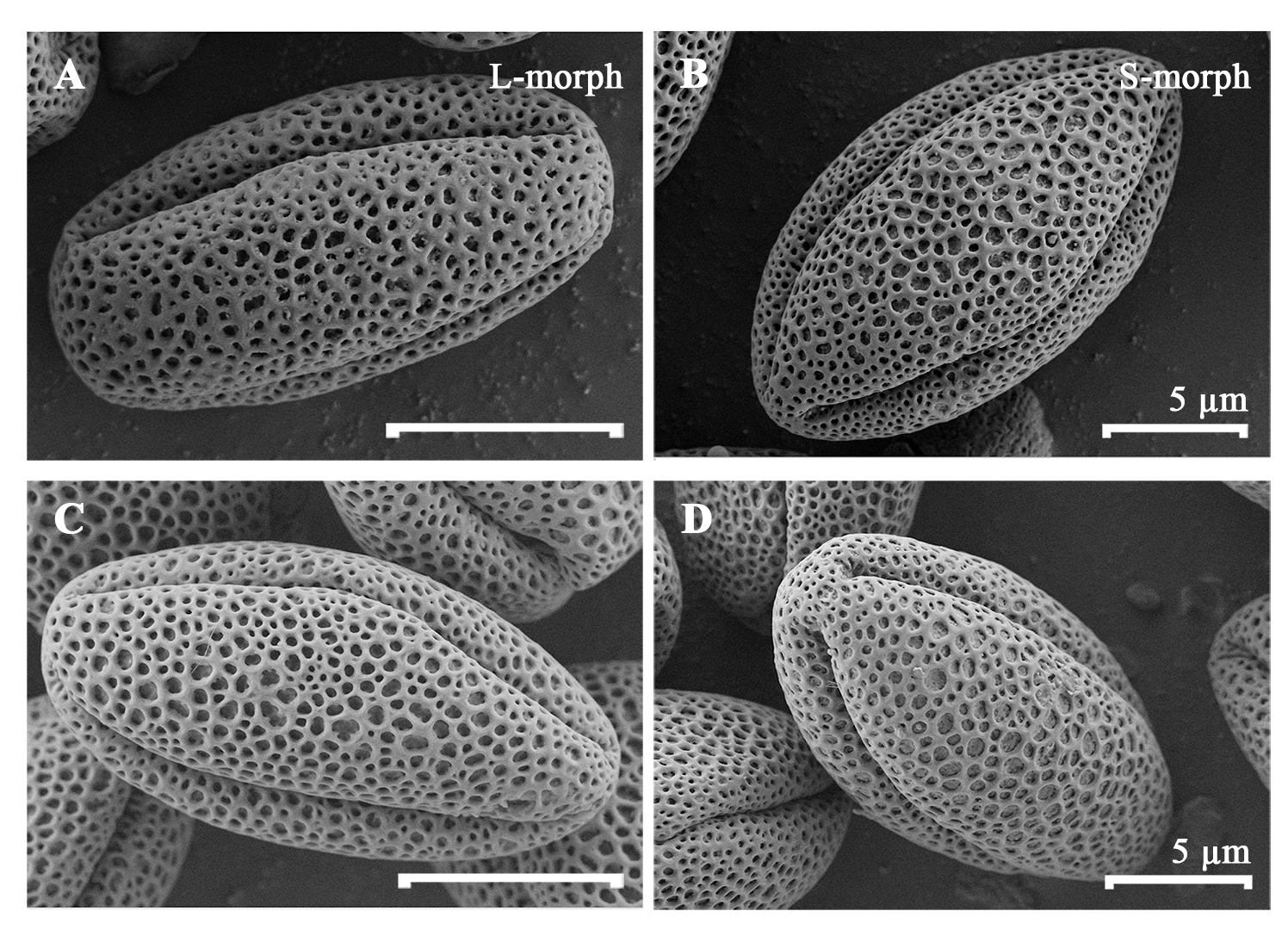


| **Appendix S3.** The significantly different analysis of pollen viability among L-,  S- and H-morphs of *Primula forbesii*. | | | | | | |
| --- | --- | --- | --- | --- | --- | --- |
| Pairs | *P* values | | | | | |
|  | 0 d | 2 d | 4 d | 6 d | 10 d | 14 d |
| L-morph vs. H-morph | **< 0.001** | **< 0.001** | **< 0.001** | **< 0.001** | **< 0.001** | 0.099 |
| H-morph vs. S-morph | **< 0.001** | **< 0.001** | **< 0.001** | 0.445 | **< 0.001** | **0.033** |
| L-morph vs. S-morph | 0.548 | 0.628 | **< 0.001** | **< 0.001** | **< 0.001** | **< 0.001** |
| Significantly different *P* values (*P* < 0.05) based on generalize linear model (GLM) are indicated in bold. | | | | | | |

**Appendix S4.** Pollen germination of L- (A), S- (B) and H-(C) morphs flowers of *Primula forbesii* after cultivating in medium for 4 h. All bars = 100 µm.


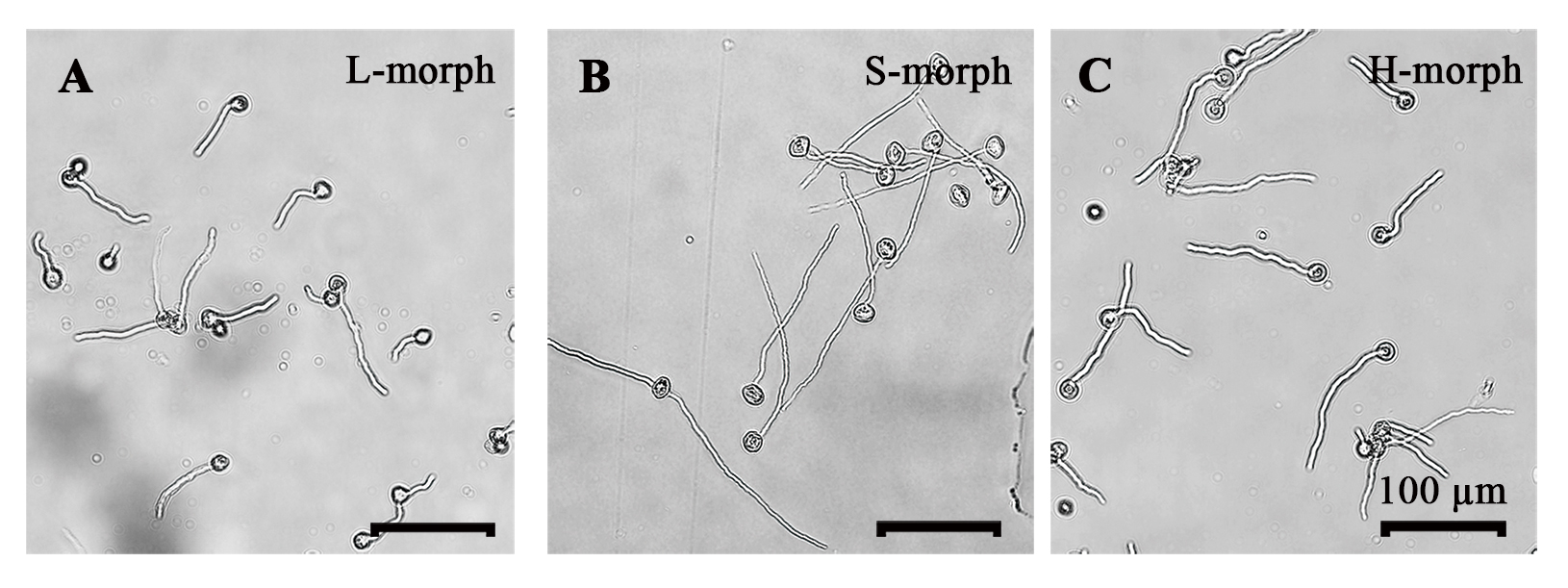


| **Appendix S5.** Mean (±SE) relative amounts of floral scent compounds in three flower morphs of *Primula forbesii.* | | | | | | |
| --- | --- | --- | --- | --- | --- | --- |
| No. | Compound | R.T. ^1^ | R.I.^2^ | L-morph (N=5) | S-morph (N=5) | H-morph (N=5) |
|  | Terpenoids |  |  | 43.15 ± 1.61 | 52.06 ± 1.92 | 23.21 ± 0.93 |
| 1 | α-Pinene | 3.722 | 1017 | 1.09 ± 0.10 | 2.86 ± 0.29 | 0.51 ± 0.22 |
| 2 | β-Pinene | 5.536 | 1102 | 1.17 ± 0.26 | 3.96 ± 0.74 | 0.46 ± 0.11 |
| 3 | Dipentene | 7.319 | 1175 | 0.12 ± 0.03 | 0.20 ± 0.05 | 0.03 ± 0.00 |
| 4 | 1,8-Cineole | 7.663 | 1190 | 1.87 ± 0.71 | 1.77 ± 0.25 | 1.28 ± 0.56 |
| 5 | Ocimene | 8.477 | 1226 | 1.01 ± 0.31 | 1.79 ± 0.42 | 1.14 ± 0.62 |
| 6 | Linalool | 13.878 | 1539 | 18.19 ± 2.00 | 13.23 ± 0.93 | 7.66 ± 0.75 |
| 7 | beta-elemene | 14.467 | 1580 | 2.35 ± 0.79 | 3.09 ± 0.52 | 2.00 ± 0.36 |
| 8 | Menthol | 15.144 | 1631 | － | － | － |
| 9 | (-)-alpha-Gurjunene | 15.47 | 1665 | 1.90 ± 0.33 | 4.45 ± 1.72 | 0.65 ± 0.10 |
| 10 | alpha - Terpineol | 15.873 | 1686 | 5.91 ± 0.81 | 9.33 ± 0.67 | 2.11 ± 0.33 |
| 11 | β-Selinene | 15.995 | 1696 | 5.71 ± 0.70 | 7.45 ± 0.30 | 4.28 ± 0.60 |
| 12 | (+)-valencene | 16.15 | 1708 | 1.31 ± 0.29 | 2.23 ± 0.23 | 0.89 ± 0.15 |
| 13 | Geraniol | 17.807 | 1845 | 0.06 ± 0.04 | 0.09 ± 0.04 | 0.01 ± 0.01 |
| 14 | Nerolidol | 19.852 | 2027 | 0.55 ± 0.24 | 1.64 ± 0.33 | 1.07 ± 0.40 |
| 15 | Perhydrofarnesyl Acetone | 20.811 | 2117 | 1.91 ± 0.78 | 1.03 ± 0.19 | 1.12 ± 0.35 |
|  | Benzenoids |  |  | 42.28 ± 2.41 | 42.98 ± 1.99 | 44.23 ± 1.32 |
| 16 | methylbenzene | 3.839 | 1023 | 0.13 ± 0.05 | 0.38 ± 0.11 | 0.04 ± 0.02 |
| 17 | ethylbenzene | 5.749 | 1111 | 0.04 ± 0.02 | － | 0.03 ± 0.01 |
| 18 | para-xylene | 5.92 | 1118 | － | － | 0.04 ± 0.01 |
| 19 | m-Xylene | 6.038 | 1123 | 0.02 ± 0.02 | － | 0.03 ± 0.01 |
| 20 | ortho-xylene | 7.083 | 1165 | 0.07 ± 0.04 | 0.02 ± 0.00 | 0.03 ± 0.03 |
| 21 | Styrene | 8.829 | 1243 | 0.15 ± 0.03 | 0.05 ± 0.01 | 0.11 ± 0.03 |
| 22 | 1,2,4,5-Tetramethylbenzene | 11.849 | 1405 | 0.01 ± 0.01 | 0.01 ± 0.01 | 0.01 ± 0.00 |
| 23 | 4-Methylanisole | 12.166 | 1425 | 4.48 ± 2.16 | 6.98 ± 1.24 | 3.77 ± 1.03 |
| 24 | Benzaldehyde | 13.486 | 1512 | 3.04 ± 0.30 | 2.95 ± 0.46 | 4.02 ± 0.53 |
| 25 | Methyl benzoate | 14.886 | 1611 | 0.98 ± 0.14 | 0.37 ± 0.03 | 1.75 ± 0.26 |
| 26 | Phenylacetaldehyde | 15.158 | 1632 | 1.36 ± 0.43 | 0.22 ± 0.07 | 1.56 ± 0.35 |
| 27 | Acetophenone | 15.263 | 1640 | 1.55 ± 0.28 | 2.69 ± 0.60 | 2.16 ± 0.18 |
| 28 | Salicylaldehyde | 15.625 | 1668 | 0.04 ± 0.01 | 0.02 ± 0.01 | 0.04 ± 0.00 |
| 29 | Benzyl Acetate | 16.273 | 1718 | 1.52 ± 0.26 | 2.02 ± 0.13 | 1.53 ± 0.14 |
| 30 | Naphthalene | 16.453 | 1733 | 0.13 ± 0.02 | 0.05 ± 0.01 | 0.09 ± 0.01 |
| 31 | Methyl salicylate | 16.89 | 1768 | 6.01 ± 1.84 | 1.86 ± 0.30 | 5.14 ± 0.73 |
| 32 | 2-Chlorophenol | 17.687 | 1834 | 0.02 ± 0.00 | 0.01 ± 0.00 | 0.02 ± 0.00 |
| 33 | 2-Methylnaphthalene | 17.784 | 1843 | 0.03 ± 0.01 | 0.01 ± 0.00 | 0.02 ± 0.01 |
| 34 | 2-Methoxyphenol | 17.821 | 1846 | 1.14 ± 0.27 | 0.55 ± 0.11 | 1.54 ± 0.41 |
| 35 | Benzyl alcohol | 17.992 | 1860 | 7.07 ± 1.29 | 9.51 ± 1.13 | 8.93 ± 1.20 |
| 36 | Phenethyl alcohol | 18.408 | 1896 | 3.12 ± 0.65 | 2.83 ± 1.13 | 2.19 ± 0.61 |
| 37 | 2,6-Di-tert-butyl-4-methylphenol | 18.49 | 1903 | 0.11 ± 0.04 | 0.03 ± 0.01 | 0.02 ± 0.01 |
| 38 | Benzeneacetonitrile | 18.611 | 1914 | 9.34 ± 1.65 | 10.22 ± 1.48 | 9.20 ± 0.91 |
| 39 | Benzothiazole | 18.973 | 1946 | 0.07 ± 0.04 | 0.02 ± 0.00 | 0.04 ± 0.01 |
| 40 | O-Cresol | 19.379 | 1983 | 0.06 ± 0.01 | 0.02 ± 0.00 | 0.05 ± 0.01 |
| 41 | Phenol | 19.403 | 1985 | 0.11 ± 0.04 | 0.04 ± 0.00 | 0.12 ± 0.02 |
| 42 | p-Anisaldehyde | 19.688 | 2011 | 0.52 ± 0.11 | 0.38 ± 0.07 | 0.49 ± 0.13 |
| 43 | p-Cresol | 20.218 | 2060 | 0.38 ± 0.16 | 0.22 ± 0.07 | 0.23 ± 0.09 |
| 44 | Eugenol | 21.161 | 2151 | 0.01 ± 0.00 | 0.01 ± 0.00 | 0.02 ± 0.01 |
| 45 | 4-Methoxybenzyl alcohol | 22.218 | 2256 | 0.55 ± 0.07 | 0.79 ± 0.17 | 0.71 ± 0.20 |
| 46 | Indole | 23.819 | 2426 | 0.20 ± 0.11 | 0.69 ± 0.32 | 0.26 ± 0.11 |
| 47 | Benzophenone | 24.179 | 2466 | 0.04 ± 0.01 | 0.02 ± 0.00 | 0.04 ± 0.02 |
|  | Fatty Acid Derivatives |  |  | 14.56 ± 2.61 | 4.96 ± 0.26 | 32.57 ± 2.01 |
| 48 | 2,3-Butanedione | 3.119 | 977 | 0.05 ± 0.02 | 0.01 ± 0.00 | 0.14 ± 0.06 |
| 49 | cis-3-Hexenal | 6.328 | 1135 | 1.03 ± 0.17 | 0.21 ± 0.05 | 1.75 ± 0.42 |
| 50 | 1-Penten-3-ol | 7.283 | 1173 | 1.01 ± 0.44 | 0.80 ± 0.10 | 1.52 ± 0.47 |
| 51 | trans-2-Hexenal | 8.066 | 1206 | 3.08 ± 0.85 | 0.57 ± 0.14 | 7.66 ± 1.83 |
| 52 | Tetramethylene sulfone | 8.203 | 1215 | 0.68 ± 0.30 | 0.48 ± 0.09 | 2.62 ± 0.70 |
| 53 | Octanal | 9.547 | 1278 | 0.01 ± 0.01 | － | 0.01 ± 0.01 |
| 54 | Hexyl acetate | 9.681 | 1284 | 0.09 ± 0.02 | 0.02 ± 0.01 | 0.05 ± 0.02 |
| 55 | cis-2-Penten-1-ol | 10.303 | 1318 | 1.25 ± 0.42 | 0.50 ± 0.13 | 2.57 ± 0.61 |
| 56 | 1-Hexanol | 10.872 | 1350 | 0.89 ± 0.35 | 0.25 ± 0.03 | 1.28 ± 0.28 |
| 57 | cis-3-Hexen-1-ol | 11.394 | 1380 | 5.17 ± 1.23 | 1.43 ± 0.10 | 13.72 ± 1.52 |
| 58 | 2-Butoxyethanol | 11.684 | 1395 | 0.02 ± 0.01 | － | 0.02 ± 0.01 |
| 59 | Acetic Acid | 12.373 | 1439 | 0.53 ± 0.11 | 0.18 ± 0.05 | 0.41 ± 0.05 |
| 60 | trans, trans-2,4-Heptadienal | 12.965 | 1477 | 0.14 ± 0.06 | 0.07 ± 0.02 | 0.21 ± 0.04 |
| 61 | 2-Ethyl hexanol | 13.033 | 1481 | 0.03 ± 0.02 | 0.03 ± 0.01 | 0.09 ± 0.03 |
| 62 | 2-Nonanol | 13.104 | 1486 | 0.03 ± 0.01 | 0.02 ± 0.01 | 0.02 ± 0.01 |
| 63 | Decyl aldehyde | 13.194 | 1492 | 0.03 ± 0.01 | 0.01 ± 0.01 | 0.01 ± 0.01 |
| 64 | Octanol | 14.016 | 1549 | 0.05 ± 0.02 | 0.05 ± 0.01 | 0.11 ± 0.04 |
| 65 | Isobutyric acid | 14.107 | 1555 | － | － | － |
| 66 | Butyric Acid | 14.919 | 1614 | － | － | 0.01 ± 0.00 |
| 67 | Caproic acid | 17.6 | 1827 | 0.03 ± 0.01 | 0.01 ± 0.00 | 0.04 ± 0.01 |
| 68 | α-ionone | 17.798 | 1844 | － | － | 0.06 ± 0.03 |
| 69 | 1-Undecanol | 18.157 | 1874 | 0.01 ± 0.00 | 0.02 ± 0.01 | 0.02 ± 0.01 |
| 70 | β-Ionone | 18.799 | 1931 | 0.35 ± 0.18 | 0.26 ± 0.08 | 0.20 ± 0.05 |
| 71 | Lauryl alcohol | 18.861 | 1936 | 0.05 ± 0.02 | 0.03 ± 0.01 | 0.03 ± 0.01 |
| 72 | Nonanoic acid | 21.086 | 2144 | 0.01 ± 0.00 | － | 0.01 ± 0.00 |
| 73 | Capric acid | 22.137 | 2249 | 0.01 ± 0.00 | 0.01 ± 0.00 | － |
| 74 | Lauric acid | 24.109 | 2458 | 0.01 ± 0.00 | 0.01 ± 0.00 | － |
| ^1^ R.T., retention time;^2^ R.I., Retention index;all data are presented as mean ± standard error (n = 5); "－"Not detected or not existed | | | | | | |

**Appendix S6.** Total ion chromatograms of floral scent compounds collected from L- (A), S- (B) and H-morph (C) in *Primula forbesii*. Numbers match the floral scent compounds in Appendix S5.


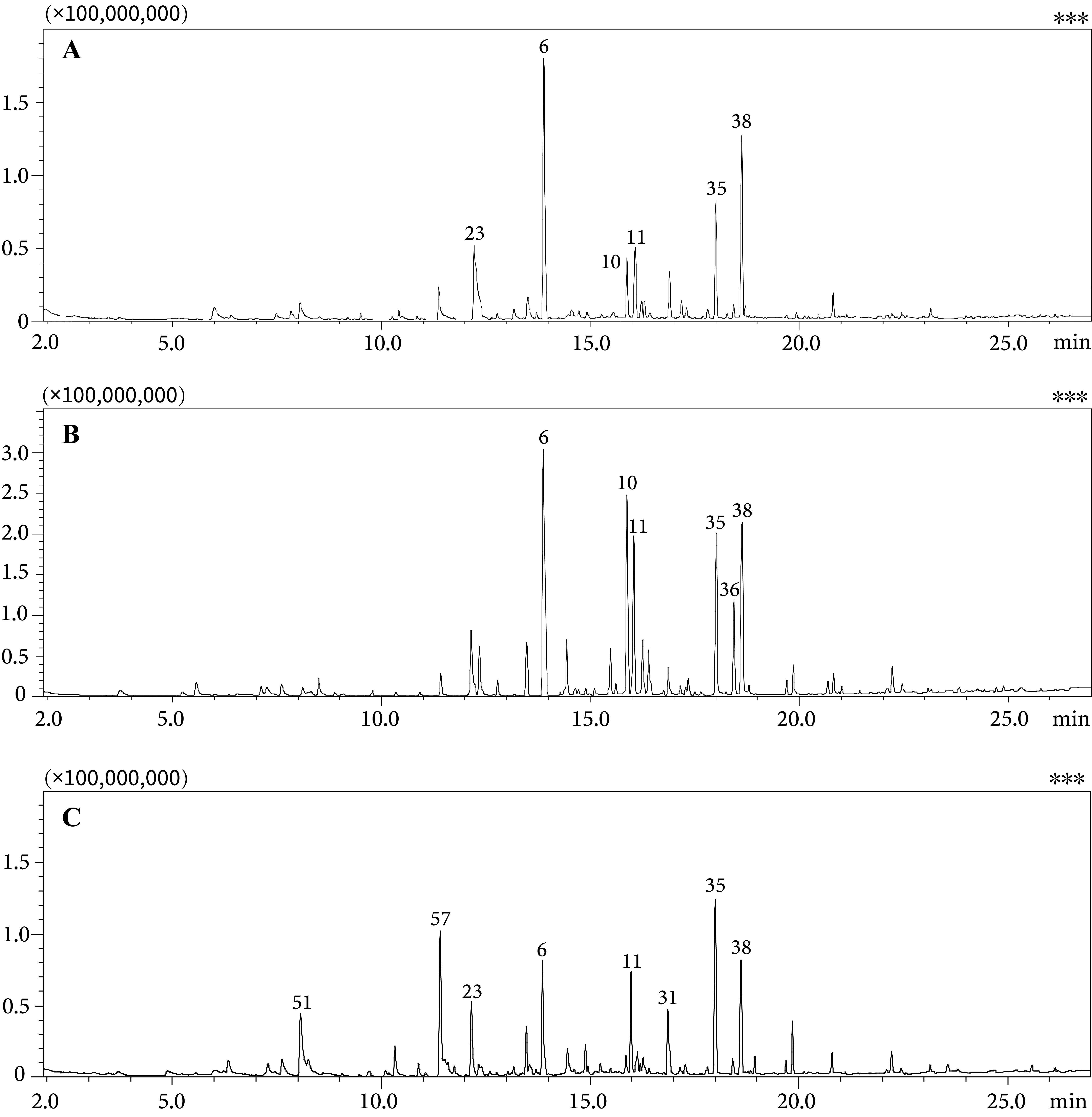

Supplement: Supplementary file 1 — Appendix S1. [file ECE3-12-e9515-s001.docx]
